# 902. From Shadowing to Success: Improving Nurse Transition Rates in the Trauma Burn Intensive Care Unit

**DOI:** 10.1093/jbcr/irag033.398

**Published:** 2026-04-06

**Authors:** Lauren A Caballero, Laura E Carpenter

**Affiliations:** Warden Burn Center at Orlando Health Orlando Regional Medical Center, Orlando, FL; Warden Burn Center at Orlando Health Orlando Regional Medical Center, Orlando, FL

## Abstract

**Introduction:**

The Trauma Burn Intensive Care Unit (TBICU) is a stressful and intense environment requiring highly skilled nurses who fit its unique culture. One-hour interviews have proven insufficient in assessing candidates. Our TBICU nurses expressed frustration over the high turnover and poorly matched hires. The purpose for this initiative was to improve Initial screening process to identify qualities indicative of success for integration into our TICU nursing culture.

**Methods:**

Pre-Interview.

In conjunction with our Unit Practice Council, we have developed a criterion for potential candidates. The candidate criteria were disseminated to our nursing recruiter in April 2025 to aid their process. After meeting established candidate criteria, a 15–30-minute phone screening was conducted by unit leadership to ensure requirements are met. Shadowing was introduced for internal candidates in April 2024 and extended to external applicants in April 2025. Shadowing enables non-burn clinicians to experience burns prior to committing to the unit. Shadowing entails the candidate nurse following a senior, precepting nurse for 3-4 hours. Candidates prioritized seeing burn dressings as part of shadow.

*Interview*.

The nurses on the unit during the shadow attended the candidate interview to discuss candidate ability to engage and integrate with the team. Peer interviews and behavioral questions remain standard.

*Post-Interview*.

In July 2025, a two-week side-by-side onboarding period began, allowing preceptors to work alongside new hires during their first six shifts off orientation.

**Results:**

Since implementing a new hiring process, the nurse retention rate has improved from 70% to 85%. The original 70% was based on 7 nurse hires between April 2024 and April 2025, while the improved 85% retention reflects 7 nurse hires from April 2025 to September 2025. All but one of the recent hires have successfully completed orientation and remained employed. Additionally, this initiative has encouraged greater bedside staff participation in peer interviews, enhancing team engagement and ownership in hiring decisions.

**Conclusions:**

The process improvement aims to add value back to the bedside staff's time. This should support positive feelings toward new hires so nurses can feel more invested, which contrasts with previous frustrations over poor culture fit and high turnover.

**Applicability of Research to Practice:**

Future data collection will assess retention rates over a longer period to evaluate the effectiveness of these changes, particularly with new burn nurses.

**Funding for the study:**

N/A.

